# Recent Advances in the Surgery of the Blood-Vessels

**Published:** 1906-12-08

**Authors:** Ernest W. Hey Groves

**Affiliations:** Assistant Surgeon to the Britsol General Hospital, and Demonstrator of Anatomy at the Bristol Medical School


### Hospital Clinics.

### RECENT ADVANCES IN THE SURGERY OF THE BLOOD-VESSELS.

By ERNEST W. HEY GROVES, M.S., F.R.C.S., Assistant Surgeon to the Britsol General Hospital, and Demonstrator of Anatomy at the Bristol Medical School.

This subject will be discussed under the following heads:—

A. Surgery of the arteries:—Ligature in continuity. Matas operations for aneurism. Direct suture of wounded arteries. Treatment of aneurisms by the introduction of wire and electrolysis. The gelatine injection of aneurisms.

B. Surgery of the veins:—The relation of thrombosis to blood platelets; lime salts; milk diet; carbonic acid. The incidence of thrombosis. The treatment of thrombosis.

*Ligature of arteries in their continuity.*—Much has happened since the old days of twenty years ago, when arteries were tied with septic ligatures whose ends were left hanging out of the wound. Now the essentials of success in ligaturing an artery are known to be three. The ligature must be tied properly, it must be aseptic, and it must not be absorbed too quickly. For the first essential it is best to employ a double strand of ligature, the first turn in the knot being tied separately in the two strands, and the second turn of the knot being tied by twisting the two strands together. This stay-knot prevents the first twist being loosened whilst the second turn is being tied. The nature of the best ligature is still open to a difference of opinion. On the one hand, silk is strong and easily sterilised by boiling, but it is practically unabsorbable. On the other hand, catgut and other animal ligatures are absorbed, but the method of sterilisation always involves reliance being placed on the exact carrying out of a complicated process. Probably the silk ligature is the one preferred by most surgeons for the occlusion of large vessels. It is also recognised that the best results will be obtained by drawing the knot tightly enough to occlude the lumen of the artery without rupturing the inner coats of the vessel.

*Matas operations for aneurism.*—But it is also being increasingly recognised that the certainty of success of a ligature of an artery for aneurism depends on its proximity to the affected part, and that any advantage gained by Hunter's operation at a distance from the sac as being concerned with a more healthy part of the artery is more than counterbalanced by the uncertainty that exists when one or more collateral vessels intervene between the ligature and the aneurism. The most modern operation for external aneurism is one in which, after the use of a tourniquet, the sac is opened freely and emptied of clot. In the case of a sacculated aneurism the mouth of the sac is then sewn together by fine catgut stitches and the walls of the aneurism doubled down as a kind of pad over the outside of the vessel. Thus the lumen of the artery is unobliterated. In fusiform aneurism the mouths of the artery as it enters and leaves the sac are found and sewn together from within the sac.

*Direct suture of wounded arteries.*—These operations of Matas are founded on the principle that the walls of blood-vessels can be sutured and will unite just like any other tissue. And this fact is now made use of in dealing with punctured wounds of large arteries. The old dictum that no wound of an artery can be considered as efficiently treated unless the lumen of the vessel is occluded has thus been given up. When a stab in the groin has opened the femoral artery the vessel has been exposed, and the opening in its wall carefully sewn together by catgut stitches and the circulation then allowed to continue in the main vessel. A still more daring advance has reached an experimental stage with promise of success. The leg of a dog has been amputated and then re-fixed to the stump, the bone wired to bone, the vein sewn to vein, artery to artery, and nerve to nerve. And it is therefore quite possible to unite together the cut ends of a completely divided artery or to open an artery in which an aseptic embolus has recently been caught, and after removing the embolus to sew up the vessel.

*The treatment of aneurisms by the introduction of wire and by electrolysis.*—Internal aneurisms can be made to consolidate by the introduction into their cavity of a length of fine wire. But of fourteen cases of abdominal aneurism in which this was tried prior to 1900 only three survived. But a good deal of the uncertainty and difficulty of the operation have been overcome by the invention of Colt's apparatus, in which a collapsible cage of wire is pushed into the sac of the aneurism through a trocar. When released from the trocar the wire cage assumes a spherical shape in the sac, and there is no danger of its protruding into the main lumen of the vessel. If, in addition to the introduction of the wire into the sac, an electric current of 40-80 milliampères be passed through it, by connecting the wire with the positive pole of a battery and a pad on the patient's back with the negative, then coagulation takes place more rapidly and more firmly. But this requires a long operation lasting from three quarters to one and a half hours. Twenty-three cases have been treated by this method, seventeen thoracic and six abdominal. Four have been cured, three thoracic and one abdominal. Nine cases showed amelioration of symptoms with prolongation of life.

*The gelatine injection treatment of aneurisms.*—If a 1 or 2 per cent. solution of gelatine in normal saline be injected into the subcutaneous tissue it is absorbed, and the coagulability of the blood is so much raised that an aneurism will often be cured by coagulation. It is necessary to inject 100 c.cm. at a time and to repeat the injection every ten to twelve days for about twelve to fifteen injections. The utmost care must be taken to sterilise the gelatine solution by placing it for an hour at a time in a steamer for three consecutive days. The results from this method do not seem to be as encouraging as their author promised when he published five cases, three of which had been cured. And two cases have died of tetanus owing to a contamination of the gelatine.

*On thrombosis, its relation to blood platelets.*—There are two main factors concerned in the production of coagulation of the blood in the living vessels. One is the condition of the blood itself, the other the condition of the wall of the vessel. And among the many varying elements in the blood itself, the platelets are now regarded as having an important influence in the process of coagulation. In the occurrence of thrombosis the platelets are the first elements which collect upon the vessel wall, and the fibrin filaments spread chiefly from the plate masses as centres. In perfectly fresh blood the platelets are round or oval masses one quarter the diameter of the red corpuscles. But they rapidly undergo changes when the blood is shed, which make them irregular and more refractile and run together in masses. They are estimated to number about 700,000 per c. centimetre. Their number is directly proportionate to that of the red cells. It has been found that if the blood of a living animal be deprived of its fibrin one portion at a time, and then returned to the circulation, it will at length be free from fibrin and uncoagulable. At this point the platelets are quite absent, the leucocytes much diminished, and the red discs slightly.

*Thrombosis in its relation to lime salts and milk diet.*—It has been shown that the presence of calcium salts is essential to the occurrence of coagulation, and that in their absence clotting will not take place; and, on the other hand, an excess of calcium salts causes such a predisposition to coagulation that thrombosis often is the result. These facts are of great importance both in prevention of hæmorrhage and in the prevention of thrombosis. For if in conditions, such as chronic jaundice, which pre-dispose to hæmorrhage, calcium chloride be administered in doses of thirty grains three times a day for several days before an operation, the tendency to post-operative hæmorrhage will be counteracted. Conversely thrombosis may be prevented by giving similar doses of citric acid, which removes the calcium salts from the blood. It has long been recognised that the convalescent stage of typhoid fever and many other asthenic conditions are often complicated by thrombosis. And it can be shown that the coagulability of the blood is much increased in these circumstances, and it also contains twice the normal amount of lime salts. These facts suggest that the liability to thrombosis after typhoid fever is due to an excess of lime salts in the blood, and this excess is probably due to the milk upon which the patients have fed so long. Cows' milk contains 1 part in 600 of lime, as compared with 1 in 800 in lime water. If this is established it would be easy to decalcify the milk by adding thirty grains of citrate of soda to each pint.

*The relation of thrombosis to carbonic acid.*—Intra-vascular coagulation brought about by the injection of foreign substances, such as snake venom or nucleo-proteid, into the circulation of animals takes place chiefly in the venous system, and is greatly favoured by an excess of carbonic acid in the blood. Chlorotic blood, by reason of its poverty in hæmoglobin, is certainly deficient in oxygen and is probably overloaded with carbonic acid. And by an inhalation of carbonic acid gas the coagulability of the blood may be greatly increased, and this has in one instance afforded an effective treatment for the bleeding in hæmophilia.

*On the incidence of thrombosis.*—As a complication of appendicitis and other abdominal diseases thrombosis is well recognised. In 3,334 cases of appendicitis reported from five London hospitals twenty-nine had thrombosis. And in all cases of peripheral thrombosis there is a great preference for the left lower limb. This may be due to the anatomical fact that the left common iliac vein is crossed almost at right angles by the left internal iliac artery and also by the right common iliac artery, whereas the right vein gently winds round its companion artery. The pressure of a loaded sigmoid colon may also have something to do with the matter.

*The treatment of thrombosis.*—Of the first importance is rest, which must generally be rigidly enforced for six weeks. In ordering the diet it is well to bear in mind the conditions which prevent coagulation and those which favour it, as shown in the following table:—

| *Coagulability increased by* | *Coagulability diminished by* |
|---|---|
| Carbonic acid. | Oxygen. |
| Lime salts. | Alcohol. |
| Milk. | Diminution of lime salts. |
| Magnesium carbonate. | Citric acid. |
| Restriction of fluid. | Rhubarb. |
| | Acid, fruits, and wines. |
| | Large quantities of fluid. |
| | Restriction of food. |
| | Tobacco smoking. |

When the patient gets up, the limb should be carefully bandaged, in order to control the excessive dilatation of the peripheral veins which is apt otherwise to take place. At the end of three months massage should be begun to diminish the œdema and to dilate the deep intra-muscular venous channels which have remained unobliterated by the thrombosis. An elastic stocking should be worn for more than a year, and if necessary a pad over the groin to prevent dilatation of the superficial veins of the abdomen.

